# Sero-prevalence of visceral leishmaniasis and its associated factors among asymptomatic individuals visiting Denan health center, southeastern Ethiopia

**DOI:** 10.1186/s40794-023-00196-8

**Published:** 2023-07-10

**Authors:** Ahmed Ismail, Solomon Yared, Sisay Dugassa, Adugna Abera, Abebe Animut, Berhanu Erko, Araya Gebresilassie

**Affiliations:** 1 College of Veterinary Medicine, Jigjiga University, Jigjiga, Ethiopia; 2 Department of Biology, Jigjiga University, Jigjiga, Ethiopia; 3 Aklilu Lemma Institute of Pathobiology, Addis Ababa University, Addis Ababa, Ethiopia; 4 Malaria and Neglected Tropical Diseases Research Team, Ethiopian Public Health Institute, Addis Ababa, Ethiopia; 5 Department of Zoological Sciences, Addis Ababa University, Addis Ababa, Ethiopia

**Keywords:** Denan, Sand fly, Sero-prevalence, Risk factor, Visceral leishmaniasis

## Abstract

**Background:**

In the Somali region of Ethiopia, visceral leishmaniasis (VL) is a public health concern. However, VL epidemiology and sand fly vectors have not been well studied in various areas of the regional state, including Denan district. Therefore, this study was conducted to determine the sero-prevalence, associated factors, and distribution of sand fly vectors of VL in Denan district, south-eastern Ethiopia.

**Methods:**

A facility-based cross-sectional study was conducted from April to September 2021 among VL patients with classic signs and symptoms visiting Denan Health Center in south-eastern Ethiopia. Using a convenience sampling method, 187 blood samples were collected from individuals who visited Denan Health Center during the study period. Blood samples were subjected to Direct Agglutination Test for the detection of antibodies to VL. A pre-tested structured questionnaire was also used to gather information on risk factors and other characteristics of knowledge and attitude assessment. Sand flies were also collected from indoor, peri-domestic, mixed forest, and termite mounds using light and sticky traps to determine the fauna and abundance.

**Results:**

The overall sero-prevalence rate was 9.63% (18/187). The sero-prevalence was significantly associated with outdoor sleeping (OR = 2.82), the presence of damp floors (OR = 7.76), and sleeping outdoor near animals (OR = 3.22). Around 53.48% of the study participants had previously heard about VL. Study participants practiced different VL control methods, including bed nets (42%), insecticide spraying (32%), smoking plant parts (14%), and environmental cleaning (8%). In total, 823 sand fly specimens, comprising 12 species in two genera (*Phlebotomus* and *Sergentomyia*), were trapped and identified. The most abundant species was *Sergentomyia clydei* (50.18%), followed by *Phlebotomus orientalis* (11.42%). Also, a higher proportion of *P*. *orientalis* was found in termite mounds (65.43%), followed by mixed forest (37.8%) and peri-domestic (20.83%) habitats.

**Conclusion:**

The study demonstrated a 9.63% sero-positivity of VL and a remarkable gap in knowledge, attitude, and practices towards VL. *P. orientalis* was also detected, which could be a probable vector in this area. Thus, public education should be prioritized to improve the community’s awareness of VL and its public health impact. In addition, detailed epidemiological and entomological studies are recommended.

## Background

Visceral leishmaniasis (VL), also known as Kala-azar, is a vector-borne parasitic disease caused by protozoan parasites of the *Leishmania donovani* complex and transmitted by blood-sucking sand flies. Clinical signs and symptoms associated with VL include fever for more than two weeks, fatigue, weakness, loss of appetite and weight, malaise, cough, enlargement of lymph nodes, spleen, and liver, and bone marrow suppression with pancytopenia [1]. It is endemic in 75 countries, and the estimated annual global incidence is 50,000–90,000 new cases [2]. About 90% of the global burden for VL is found in just 7 countries, 4 of which are in Eastern Africa (Sudan, South Sudan, Ethiopia, and Kenya), 2 in Southeast Asia (India, Bangladesh), and Brazil, which carries nearly all of the cases in South America [3, 4].

Ethiopia has reported the third-largest number of VL cases (1990), following South Sudan (2840) and Sudan (2813) of any country in the sub-Saharan Africa region [5]. In Ethiopia, cases of VL have been reported from six regions (Tigrai, Amhara, Oromia, Southern Nations and Nationalities People’s Region, Somali and Afar) [6, 7], with an annual burden estimated to be between 4, 500 and 7, 400 cases [8]. The incidence rate per 10,000 people in endemic areas is 6.28 [4]. This systemic disease is known to be endemic in the Metema and Humera plains in the northwest; in several localities of south western Ethiopia, i.e., the Omo plains, the Aba Roba focus in Segen valley, and the Woito River valley adjacent to South Omo; in southern Ethiopia around the Moyale area close to the borders with North Kenya; and in south eastern Ethiopia around the Genale river basin in Oromia Regional State and the Afder and Liban zones in Somali Regional State [7].

In the Somali Regional State, VL outbreaks were first reported in 2001 from the Afder, Liben, Denan, and Hagele areas, bordering Kenya and Somalia [9]. Subsequently, VL cases have been reported sporadically from different areas of the regional state [10, 11]. What is more, the national risk map survey indicated that the wide geographical area stretching from the south-eastern part of the Somali region to the Ethio-Somali-Kenyan boundaries is a high-risk area for VL [12].

Studies involving knowledge, attitudes, and practices (KAP) have also been carried out in Ethiopia regarding the level of awareness about VL and associated risk factors for its transmission and control measures [13, 14]. Such studies reported that dog ownership, sleeping under an *Acacia* tree during the day, sleeping outside at night, the presence of termite hills, and poor housing conditions were risk factors for increasing VL infection. Understanding risk factors for VL is crucial for the design of appropriate interventions. However, the epidemiology and risk factors of VL in the Somali region in general and Denan district in particular have not been adequately addressed. It is also important to take into account the social, ecological, and cultural differences in this part of the country. For example, most of the communities in the Somali region are pastoralists who regularly move from place to place in search of food and water sources for their livestock. Due to religious beliefs, the wider inhabitants in the region do not keep dogs, which have been evidenced as the main risk factors for VL infection in earlier studies. Considering these issues, it is imperative to generate epidemiological information on the sero-prevalence and associated factors of VL in this focus.

So far, 65 species of sand fly belonging to the genera *Phlebotomus* and *Sergentomyia* have been identified in Ethiopia [15]. Of these, the incriminated vectors of VL from which parasites were detected include *P. martini*, *P. celiae*, and *P. orientalis* for *L. donovani* from the south, south-west, and northern foci [16–18]. Studying sand fly vectors in VL endemic foci is very important to improve our understanding of the transmission dynamics and design vector control methods to reduce the burden of the disease. An earlier entomological study carried out on the eastern part of the Somali region showed the presence of 12 sandfly species, including *P. martini* and *P. orientalis*, which are proven vectors of VL in Ethiopia [19]. However, there are wider gaps in the knowledge of habitat preferences, bionomics, and abundance of sand flies in this VL focus of the Somali region.

Therefore, the investigation was designed to determine the sero-prevalence, knowledge, attitude, and practices, and associated factors of VL, as well as the abundance of sand fly species in Denan district, south-eastern Ethiopia.

## Methods

### Study area

**Fig. 1:**
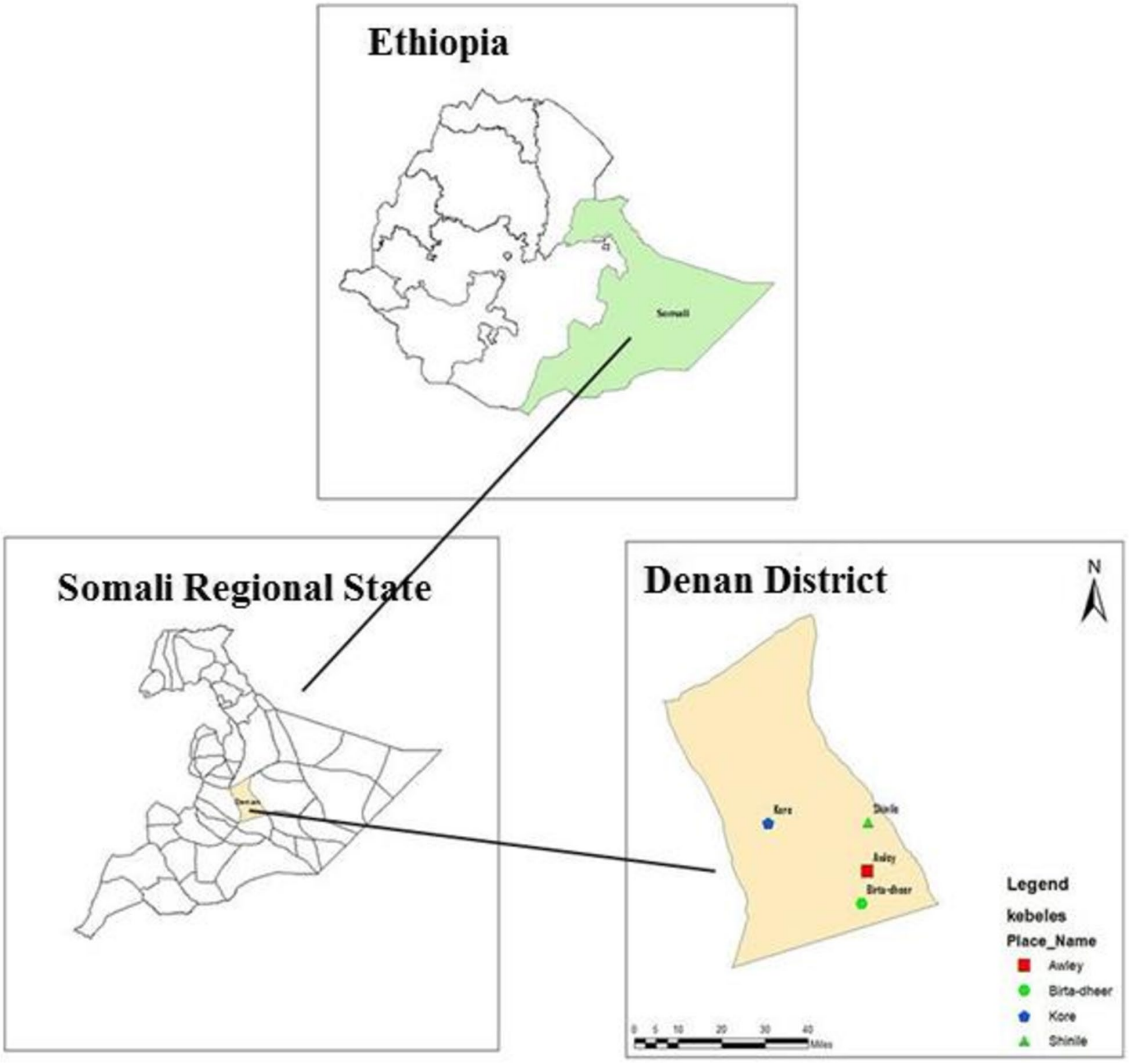
Map of the study area in the Somali Regional State, southeastern Ethiopia

The study was carried out in Denan district, south-eastern Ethiopia (Fig. 1). Denan town, the administrative center of the district, is located 1,123 kilometers southeast of Addis Ababa. It is found at an elevation of about 490 meters above sea level, with a latitude and longitude of 6° 44’ 59.99” N and 43° 19’ 60.00” E, respectively. The total population of Denan district is about 33,784 people, according to the last Ethiopian national census of 2007 [20]. The district is composed of 12 administrative *kebeles* (the smallest administrative unit). Two public health centers and nine health posts are found in Denan district, providing healthcare services to the community. Only Denan Health Center provides VL diagnostic services, and suspected patients are clinically examined.

The study area has a subtropical desert climate with a yearly temperature of 29.39 °C. It also receives about 19.24 millimeters of precipitation and has 41.53 rainy days annually. Animal husbandry is the main livelihood of villagers and mobile pastoralists. Squirrels, rodents, white-tailed mongooses (*Ichneumia* spp.), and jackals were some of the wildlife observed during our study.

Four sand fly sampling habitats such as indoor, peri-domestic, mixed forest, and termite mounds were considered. The housing type in the study area is dominantly Aqal-type, dome-shaped huts of traditional Somali houses, in which they are constructed of wooden and woven mats. The houses are situated in the peri-domestic habitats, consisting of animal enclosures, where different animals such as cattle, goats, sheep and chicken are kept. Mixed forest with scattered vegetation, mostly *Acacia-Commiphora-Boswellia* trees and bushes, and dome-shaped termite mounds surround the periphery of human residence.

### Study design and population

A facility-based cross-sectional study was conducted from April to September 2021 in Denan Health Center in the Somali Region, southeastern Ethiopia. The study participants were all patients who had been suspected of VL infection (i.e., individuals with fever for more than two weeks and an enlarged spleen (splenomegaly) and/or enlarged lymph nodes (lymphadenopathy)) and were tested for clinical examination at the time of their visit. Patients who had a previous history of VL and had been in the area in the kebeles for less than 3 months were excluded. The study participants were enrolled using a convenience sampling method.

For the KAP study, suspected patients willing to participate were included, while study participants who couldn’t communicate due to impairment, severe sickness, or mental illness and those who did not provide consent were excluded.

### Sample size

The sample size was estimated according to [21] previous prevalence of VL for serological tests, which is 15.8% [10] (considering the geographical location and similar socio-economic set-ups), with a 95% confidence interval (CI) of Z = 1.96 and a 5% degree of precision (d) using the formula *N* = [(Z)2 X P (1-P)]/d2. Consequently, a total of 204 human sera were required; however, 17 participants were excluded from the study as they declined to give blood samples.

### Blood Sample Collection

5 mL of venous blood was collected following the standard operating procedures. Blood samples were collected from the forearm veins of the study participants using sterile needles by trained clinical laboratory technicians from the Denan Health Center. Serum was separated by centrifugation from coagulated blood and stored at -20 °C at the health center. Direct Agglutination Test (DAT) was performed in the facilities of the Leishmaniasis Research and Diagnostic Laboratory at the Ethiopian Public Health Institute.

### Direct Agglutination Test (DAT)

In performing DAT, serum samples were serially diluted in physiological saline (0.9% NaCl) containing 0.8% β-mercaptoethanol. Two-fold serial dilutions of the sera were made, starting at a dilution of 1:100 and going up to a maximum serum dilution of 1:102,400. Freeze-dried DAT antigen produced by KIT Biomedical Research (DAT, Institute of Tropical Medicine—Antwerp (ITM), Belgium) was reconstituted with physiological saline. 50 L of DAT antigen solution was added to each well containing 50 µl of diluted serum. The results were read after 18 h of incubation at ambient temperature. Appropriate control samples with known DAT titres were included as controls. A sample is considered positive if it has a titre ≥ 1:1600, the cut-off value of the DAT [22].

### Assessment of KAP and associated factors

A structured questionnaire was designed to collect information regarding socio-demographics and associated factors such as house construction material and housing conditions (floor dampness, wall cracking, and roofing), domestic animal ownership, sleeping habits, use of bed nets, and individual night and day activity. The questionnaire also comprised questions relating to the respondents’ knowledge, attitudes, and common practices towards VL and sand flies. The questionnaire was first developed in English and translated into Af-Somali (the local language). Following this, it was administered to 187 study participants who gave blood for a serological test from April to September 2021. Two trained laboratory technicians from the health center were selected to collect the data. Training was given to the data collectors on how to conduct the interview, the content of the questionnaire, data quality, and ways to approach respondents.

### Data quality control

Designing proper data collection tools and training data collectors, laboratory professionals, and supervisors were measures taken to assure data quality. Before the actual data collection, the questionnaire was tested on non-selected patients. During data collection, questionnaires were reviewed and checked for completeness, accuracy, and consistency on a daily basis. In addition, the data collectors and two researchers randomly paid a visit to the houses of these patients to validate their responses in the interview and the overall appearance of their housing conditions. The VL serological test was performed according to strict standard operating procedures in a well-established facility of the Leishmaniasis Research and Diagnostic Laboratory.

### Sand fly collection and processing

Entomological investigations were undertaken in four *kebeles* (Kore, Shinile, Birta-dheer, and Awley) of Denan district during April 4–12, 2021. The above four kebeles were selected as sampling sites based on prior knowledge of sand fly ecology from other places and accessibility to transportation. In the sampling villages, four representative trapping habitats, such as indoor, peri-domestic, mixed forest, and termite mounds, were identified and used for the entire sand fly species collection. Sand flies were trapped for two consecutive nights at each sampling village, constituting 48 trap nights (i.e. 24 traps multiplied by 2 nights). Sand flies were collected using CDC light traps and sticky traps.

### CDC light traps (LTs)

LTs were deployed at peri-domestic, mixed forest, and termite mounds. Two LTs were set at representative sites of peri-domestic habitats (animal enclosure, against the house wall and inside the compound of the house). Another two LTs in each village were positioned to sample sand flies in places of mixed forest. Similarly, two LTs were suspended in termite mounds. The traps were deployed 1 h before sunset and collected at dawn the next morning. Afterwards, the sand flies were sorted by sex and genus (*Phlebotomus* or *Sergentomyia* spp.) and preserved in 70% ethyl alcohol for later species identification.

### Sticky traps (ST)

A4-sized white sticky traps of polypropylene sheets coated with sesame oil were used for capturing sand flies from all sampling habitats. The ten A4-sized STs were divided into 2 sets, each having 5 A4-sized sheets tied together on nylon string about 50 cm apart, and these were placed inside 2 different houses in each village to intercept and capture any endophilic sand flies. Similarly, 2 sets of STs were suspended randomly on cracked walls and animal enclosures in the peri-domestic environment of each village. In addition, two sets of STs were deployed separately in representative sites of mixed forest and termite mounds. Each morning, sandflies from STs were removed using forceps and stored in 96% ethyl alcohol in labeled vials for identification.

### Mounting and identification of sand flies

Sand fly specimens were dissected and mounted on microscope slides in Hoyer’s medium with their heads separated from their thoraxes and abdomens. Slide-mounted flies were then identified to species level based on the external genitalia of males and the pharynx, antennal features, and spermathecae of females, according to standard morphological keys [23].

### Data analysis

Statistical analyses were conducted using IBM SPSS statistics, version 20 for Windows (SPSS Inc., Chicago, IL, USA), and Microsoft® Office Excel 2007. Descriptive statistics was computed to determine frequency and percentage. The Chi-squared (Χ²) test was used to determine the associations between socio-demographic characteristics and VL positivity. Logistic regression was used to determine possible factors associated with VL. For all included studies, a *P* < 0.05 was regarded as statistically significant.

## Results

### Socio-demographic characteristics

In total, 187 individuals who were clinically examined for VL at Denan Health Center from April to September 2021 were enrolled in the study. Of these, 119 (63.64%) and 68 (36.36%) were males and females, respectively. The median age was 27.9 years (Interquartile range (IQR) 20–35). The majority of study subjects [110 (58.82%)] had no formal education. Most of the participants [102 (54.55%)] are pastoralists, and they had a family size of 3 (54%). A Higher proportion of participants live in Aqal (55.1%), and these houses were found close to termite mounds (48.1%) (Table 1).

**Table 1:** Socio demographic characteristics of study participants of Denan district, Somali Region, south east Ethiopia, 2021

Variable	Categories	Frequency	Percentage
Age	≤ 18	38	20.32
	19–36	112	59.89
	37–54	29	15.51
	> 54	8	4.28
Gender	Male	119	63.64
	Female	68	36.36
Marital status	Single	58	31.02
	Married	127	67.91
	Widowed	2	1.07
Family size	≤ 3	101	54
	≥ 4	86	46
Community lifestyle	Pastoralist	102	54.55
	Agro-pastoralist	23	12.3
	Urban	62	33.16
Education	Illiterate	60	32.09
	Religious education	50	26.74
	Primary	37	19.79
	Secondary school and above	40	21.39
House type (wall)	Woven mats (Aqal)	103	55.10%
	Mud and stick	43	22.90%
	Concrete blocks	41	21.90%
Termite mounds near house	Yes	90	48.10%
	No	97	51.90%
Total		187	

### Sero-prevalence and associated factors of VL

The overall sero-prevalence rate of VL in the study area was 9.63% (18/187). The sero-prevalence rates did not significantly vary between males (6.4%) and females (3.2%) (P > 0.05, Table 2). Patients in the age groups 19–36 and 37–54 had the highest DAT positivity rates of 5.9% and 2.1%, respectively, while the lowest positivity rates were observed in the age groups above 54 (0.5%), followed by less than or equal to 18 (1.1%) (Table 2). The difference in DAT positivity by age group was statistically significant (*P* < 0.05). In addition, higher DAT positivity was observed in patients who came from Danan town [7 (38.9%)], followed Birt-der [5 (22.2%)], Awley [3(16.7%)], Kore [2 (11.1%)], and Dambarwayne [1 (5.6%)].

**Table 2:** Association of socio-demographic factors with sero-prevalence rate of VL in Denan district, Somali Region, southeast Ethiopia, 2021

**Variables**	**Categories**	**Total**	**DAT positive # (%)**	***P- value***
Gender	Male	119	12 (6.4%)	0.498
	Female	68	6 (3.2 %)	
Age	≤ 18	38	2 (1.1%)	0.038
	19-36	112	11 (5.9%)	
	37-54	29	4 (2.1%)	
	> 54	8	1 (0.5%)	
Marital status	Single	58	7 (3.7%)	0.251
	Married	127	11 (5.9%)	
	Divorced	2	0	
Family size	<3	101	10 (5.3%)	0.754
	≥4	86	8 (4.3%)	
House type (wall)	Woven mats (Aqal)	103	13 (7.0%)	0.043
	Mud and stick	43	5 (2.6%)	
	Concrete blocks	41	0	
Wall characteristics	Cracked	121	14 (7.5%)	0.045
	Not cracked	66	4 (2.1%)	
Floor condition	Damp	54	1 (1.8%)	0.049
	Dry	133	17 (7.8%)	
Sleeping outside	Yes	141	16 (8.6%)	0.021
	No	46	2 (1.1%)	
Sleeping outside near animal shelter	Yes	102	14 (7.5)	0.046
	No	85	4 (2.1%)	
Sleeping near specific vegetation	Yes	30	2 (1.1)	0.4
	No	157	16 (8.5)	
Wood burning	Yes	47	4 (2.1%)	0.185
	No	140	14 (7.5%)	
House sprayed with insecticides	Yes	61	5 (2.6%)	0.249
	No	125	13 (7.0%)	

Significant differences in the sero-prevalence of VL were found among age groups, house walling type, floor condition (dampness), outdoor sleeping, and sleeping near an animal shelter (*P < 0.05*). However, variables such as sex, family size, marital status, wood burning, sleeping near specific vegetation, and house spray with insecticide were found to be statistically non-significant (*P > 0.05*) (Table 2).

Table 3 shows risk factors associated with VL in multivariable logistic regression models. Being in the age group of 19–36 increases the odds of getting a VL infection (OR = 0.76; 95%: Cl 0.086–6.78; *P* = 0.034). Similarly, individuals with the habit of sleeping outdoors near animal shelters are more likely to be at risk of acquiring a VL infection than those sleeping indoors (OR = 3.22; 95%: Cl 1.02–10.19; *P* = 0.046). Participants who owned houses with damp floors were found to have high DAT positivity compared to those who had houses with dry floors (OR = 7.76; 95%: Cl 1.01–59.90; *P* = 0.049) (Table 3).

**Table 3:** Factors associated with transmission of VL in multivariate analysis in Denan district, Somali Region, southeast Ethiopia, 2021

Variables	Categories	Total	DAT positive # (%)	Odds Ratio (OR)	95% CI		*P*- value
					Lower	Upper	
Age	≤18	38	2 (1.1%)	0.44	0.207	0.94	0.034
	19-36	112	11 (5.9%)	0.76	0.086	6.78	
	37-54	29	4 (2.1%)	1.12	0.11	11.69	
	>54 (ref)	8	1 (0.5%)	1			
Floor condition	Damp	133	17 (7.8%)	7.76	1.01	59.90	0.049
	Dry (ref)	54	1 (1.8%)	1			
Outdoor sleeping	Yes	141	16 (8.6%)	2.82	0.62	12.74	0.041
	No (ref)	46	2 (1.1%)	1			
Sleeping outdoor near animal shelter	Yes	102	14 (7.5)	3.22	1.02	10.19	0.046
	No (ref)	85	4 (2.1%)	1			

### Knowledge of VL and sand fly vectors

Among the total participants, 100 (53.48%) had heard about the disease, and 42% responded that sand fly bites are responsible for VL transmission (Table 4). The most common sources of VL-related information were health personnel (48%), followed by friends and neighbors (39%), mass media (radio and television) (5%), and school (8%). Regarding clinical signs and symptoms of VL, 48% of respondents indicated that abdominal swelling is the key symptom of VL. A substantial number of respondents (78%) mentioned that the disease is treatable. Around 87.5% of the respondents replied that they are aware of sand fly breeding habitats, and 21% indicated that sand flies have multiple breeding habitats.

**Table 4:** Knowledge on VL among study participants Denan district, Somali Region, southeast Ethiopia, 2021

Variables	Categories	Frequency	Percentage (%)
Heard about VL (*n* = 187)	Yes	100	53.48
	No	87	46.52
Source of VL information (*n* = 100)	Health personnel	48	48
	Friends and neighbors	39	39
	Media (Radio and TV)	5	5
	School	8	8
Mode of transmission (*n* = 100)	Contact with sick people	18	18
	Sand fly bite	42	42
	Spoiled food	3	3
	Mosquito bite	23	23
	Don’t know	14	14
VL signs and symptoms (*n* = 100)	Fever and abdominal swelling	48	48
	Headache and fatigue	3	3
	Multiple answers	38	38
	Don’t know	11	11
Treatability of VL (*n* = 100)	Yes	78	78
	No	7	7
	Don’t know	15	15
Identify the sand fly (*n* = 187)	Yes	96	51.33
	No	91	48.7
Sand flies found around your houses	Yes	76	79.17
	No	20	20.83
Sand fly breeding sites	Cracks of houses	20	20.8
	Termite mounds	14	14.6
	Near animal shelter	12	12.5
	Crevices of tree	17	17.7
	Multiple domestic areas	21	21.9
	Don’t know	12	12.5

### Attitudes and practices of the participants on VL and sand flies

Table 5 shows the attitudes and practices of study participants about VL. Less than 50% of the respondents stated that VL is an important public health problem in the area. The majority (68%) of respondents indicated that they prefer to seek treatment in health centers as their first priority for VL treatment. Regarding fatality, 44% of the respondents claimed that VL is fatal. Only 36% of the respondents believed that VL as a preventable disease (Table 5).

**Table 5:** Attitude and practice of the participants on VL and sand flies vectors (*n* = 100)

**Variables**	**Categories**	**Frequency**	**Percent (%)**
**Attitudes**			
Severity of VL	Very serious	3	3
	Serious	45	45
	Ordinary	52	52
VL important public health problem	Yes	48	48
	No	52	52
Preventability of VL	Yes	36	36
	No	59	59
	I don’t know	5	5
VL will be fatal if left untreated	Yes	44	44
	No	56	56
**Practices**			
Drug preference for VL treatment	Specific medicine	44	44
	Indigenous medicine	12	12
	Both specific & indigenous	36	36
	Don’t know	8	8
Seek treatment from	Health center	68	68
	Traditional medicine	20	20
	Self-medication	12	12
Prevention measures of VL	Bed net	42	42
	Insecticide spraying	32	32
	Smoking tree branches	14	14
	Cleaning environment	8	8
	Don’t know	4	4

In this study, participants were practicing different methods to prevent VL: 42% of the respondents used a bed net, 32% used insecticide spraying, 14% practiced smoking plant parts, and 8% cleaned their environments (Table 5).

### Species composition and relative abundance of sand flies

A total of 823 sand flies were collected and identified. The sand fly fauna included both the genera *Phlebotomus* and *Sergentomyia*. Twelve species were morphologically identified, and these sand fly species were *P. orientalis*, *P. alexandri*, *P. papatasi*, *P. rodhaini*, *P. saevus*, *S. clydie*, *S. bedfordi group*, *S. schwetzi*, *S. africana*, *S. antennata*, *S. squamipluris*, and *S. heisch* (Table 6).

**Table 6:** Relative abundance and fauna of sand flies collected in Denan district, Somali Region, southeast Ethiopia

Species	Type of collections methods				Total	Relative frequency (%)
	Light traps		Sticky traps			
	Male	Female	Male	Female		
*Phlebotomus orientalis*	25	28	22	19	94	11.42
*P. alexandri*	17	25	11	12	65	7.89
*P. papatasi*	11	24	5	6	46	5.59
*P. rodhaini*	1	1	3	0	6	0.61
*P. saevus*	1	2	1	0	4.5	0.47
*Sergentomyia clydie*	102	113	128	70	413	50.18
* S. bedfordi group*	14	36	17	23	90	10.94
* S. schwetzi*	5	4	16	19	45	5.35
* S. africana*	4	28	3	5	40	4.865
* S. antennata*	4	5	3	0	12	1.46
* S. squamipluris*	5	4	0	0	10	1.096
* S. heischi*	0	0	1	0	1	0.126
**Total**	189	270	210	154	823	

Of the 214 *Phlebotomus* specimens, *P. orientalis* constituted 43.9% of the sand flies captured, followed by *P. alexandri* (30.37%), *P. papatasi* (21.49%), *P. rodhaini* (2.33%), and *P. saevus* (1.86%). Among *Sergentomyia* spp., *S. clydei* was the most predominant species, accounting for 67.81% and 50.18% of *Sergentomyia* species and the entire sand fly collection, respectively. The abundance of other *Sergentomyia* species in descending order was *S. bedfordi group* (14.78%), *S. schwetzi* (7.22%), *S. africana* (6.57%), *S. antennata* (1.97%), *S. squamipluris* (1.48%), and *S. heischi* (0.16%) (Table 6).

### Habitat preferences of ***Phlebotomus*** spp

Habitat preferences of *Phlebotomus* spp. are presented in Table 7. A higher proportion of *P*. *orientalis* was found in termite mounds (65.43%), followed by mixed forest (37.8%) and peri-domestic (20.83%) habitats. However, STs placed indoors did not collect a single specimen of P. orientalis (Table 7). In contrast, a higher proportion of *P. alexandri* was trapped in mixed forest habitats (48.78%), followed by termite mounds (18.52%) and peri-domestic (16.67%). Unlike *P. orientalis*, two specimens of *P. alexandri* were captured inside houses (Table 7).

**Table 7:** Species of sand flies collected from different sampling sites of Denan district, Somali Region, southeast Ethiopia, 2021

Species	Proportion of sand fly specimens in different sampling habitats											
	**Indoor**			**Peri-domestic**			**Mixed Forest**			**Termite mounds**		
	Male	Female	Total (%)	Male	Female	Total (%)	Male	Female	Total (%)	Male	Female	Total (%)
*Phlebotomus orientalis*	0	0	0	5	5	10 (20.83)	13	18	31 (37.8)	29	24	53 (65.43)
*P. alexandri*	1	1	2 (66.67)	2	6	8 (16.67)	19	21	40 (48.78)	6	9	15 (18.52)
*P. papatasi*	0	1	1 (33.33)	9	19	28 (58.33)	4	3	7 (8.54)	3	7	10 (12.35)
*P. rodhaini*	0	0	0	0	1	1 (2.08)	4	0	4 (4.88)	0	0	0
*P. saevus*	0	0	0	0	1	2 (2.08)	0	0	0	2	1	3 (3.7)
*Total*	1	2	3	16	32	48	40	42	82	40	41	81

### Sex ratio

Sex ratios (males: females) for different sandfly species showed that females caught by all methods combined were higher than males, with an overall sex ratio of 094:1 (Table 8). In light traps, the sex ratio was 0.7, while in sticky traps; it was slightly in favor of males with 1.36:1.

**Table 8:** Sex ratio of sandfly species collected from different habitats using CDC light traps and sticky traps in Denan district

Sand fly species	Type of collection methods					Sex ratio
	Light traps		Sex ratio	Sticky traps		
	Male	Female		Male	Female	
*P. orientalis*	25	28	0.89	22	19	1.16
*P. alexandri*	17	25	0.68	11	12	0.92
*P. papatasi*	11	24	0.46	5	6	0.83
*P. rodhaini*	1	1	1	3	0	NA
*P. saevus*	1	2	0.5	1	0	NA
*S. clydie*	102	113	0.9	128	70	1.83
*S. bedfordi group*	14	36	0.39	17	23	0.74
*S. schwetzi*	5	4	1.25	16	19	0.84
*S. africana*	4	28	0.14	3	5	0.6
*S. antennata*	4	5	0.8	3	0	NA
*S. squamipluris*	5	4	1.25	0	0	NA
*S. heischi*	0	0	0	1	0	NA
**Total**	189	270	0.7	210	154	1.36

## Discussion

Wide areas in the Somali region have been predicted to have a high VL risk based on environmental factor-based geographical information and statistical risk mapping [12]. However, data on the epidemiology of VL in various endemic areas of the region barely exists. The overall sero-prevalence of *L. donovani* among asymptomatic individuals was 9.63% by DAT. In our study, the presence of antibodies against *Leishmania* was used to determine the prevalence rate of *Leishmania* infection in VL suspected individuals. However, antibodies to *Leishmania* might not be detectable in all asymptomatic subjects; therefore, it may underestimate the prevalence of the *Leishmania* infection in the present study. Importantly, due to the time lapse between specimen collection and processing procedures brought on by the research area’s distance from the laboratory at the Ethiopian Public Health Institute in Addis Ababa, the sero-prevalence estimate may also be lower. Albeit these, the DAT positivity rate of 9.63% in this survey was lower than the previous rate of 12.5% among migrant laborers in Kafta-Humera lowlands [24] and higher than that from Raya Azebo, northeastern Ethiopia [25], and from different areas of the Benishangul-Gumuz region, western Ethiopia [26], who reported 0.8% and 5.9%, respectively. In addition, the current sero-prevalence rate was higher than the previous report of 3.0% using the ELISA test in southeastern Ethiopia [11]. Possible reasons for such variation could be related to differences in agro-ecological settings, possible predisposing risk factors, serological diagnostic techniques, research designs, and intervention strategies.

Understanding the factors that determine VL exposure in endemic foci provides a vital foundation of knowledge for designing and developing effective control methods. The findings of this study showed that adults in the age group of 19–36 had elevated VL risk. In contrast to this finding, earlier studies in Ethiopia [11, 27, 28] and elsewhere [29–31] have shown a higher risk of VL in individuals under 15 years compared to adults. The contributing factors for such a higher burden of disease among adults might be related to activities like migratory and outdoor lifestyles, involving cattle herding or sleeping outside, that imply an increased potential exposure to the sand fly vector and that are culturally specific to male adolescents and male adults. The differences in design may also account for this difference, as the cited studies were carried out in a population with a different age distribution, with more than 50% of the sample being below 15 years of age.

The results also showed a strong association between VL and poor housing conditions, such as floor dampness. Floor dampness was identified as a major independent risk factor in India and Nepal [32, 33], apparently showing that houses with damp floors could provide adequate needs for the survival of sand fly vectors. The people in the study area often spray water onto the floor in order to make it humid, possibly creating a suitable microhabitat for the sand flies. Outdoor sleeping was also identified as a risk factor for VL in our study area, which is also consistent with other reports [11, 14, 28, 34]. Given that a higher proportion of sand flies were caught outdoors and most adult male people in the study area sleep outside the house to avert heating during the dry period, this apparently increases the risk of being bitten by sand fly vectors of VL. However, owning a large family size was not found to be associated with increased VL risk in our study area, which is in disagreement with earlier reports that large family size was associated with a higher risk of VL [13, 35].

The current study also assessed participants’ knowledge, attitudes, and practices about VL infection. A little more than half of the respondents (53.48%) had heard about VL. Our finding is lower than the 85% and above found in earlier reports in Ethiopia [36]. Only 42% of study participants knew that sand lies are the causes of VL transmission. This value is higher than the findings of reports on VL in northwest Ethiopia (30%) [13]. However, this result is lower than the 68.1% found in a study in northwest Ethiopia [36]. Of the respondents who had knowledge about VL, 48% reported that fever and abdominal swelling are the key symptoms of VL. Such variability of knowledge among the various studies could be associated with the differences in the setting of the study and the public awareness activities conducted. Based on the information we obtained from the local health officer, comprehensive health education campaigns are already in place in the district to enhance community awareness about various vector-borne diseases. We believe that such health education campaigns need to be customized to the local context, taking into account the ecological and socio-cultural conditions of the local community.

Most of the respondents (59%) also believed that VL is not preventable, while only 36% of the study participants said that the disease could be prevented. Our result is in contrast with Alemu et al. [36] and Berhe et al. [37], who reported that 81.2% and 90.5% of respondents believe VL is treatable, respectively. In addition, only 44% of the study participants knew that if the disease is left untreated, the outcome will be death, which is by far lower than that of a study reported from northwest Ethiopia [36], where 96.7% of participants perceived that VL is fatal. In our study area, patients have poor perceptions towards VL preventability and its fatality, which could be related to a lower level of knowledge on treatment outcomes. It is recommended that health education programs be strengthened to increase people’s awareness. For example, health extension workers can play an active role in the spread of health education and communication on the transmission and prevention of communicable diseases such as VL, where their involvement in different areas of Ethiopia has brought success in the fight against malaria [38].

In this study, it was evidenced that, on top of the bed net, other preventive and control activities were practiced by the study participants to protect themselves from any biting flies, including sand flies. 42% of the respondents use bed nets, insecticide spraying (32%), smoking tree branches (14%), and cleaning their environment (8%) for the control of VL transmission by sandfly bites. This percentage is higher than that of a study carried out in the Indian state of Bihar [39], where 21.4% of the respondents used bed nets for the control of sand fly bites. However, it is lower than a report from northwest Ethiopia, where around 70% of the respondents used a bed net for the prevention of VL [13]. In Denan district, malaria is a public health problem, and the community mainly uses long-lasting insecticide-treated bed nets (unpublished data, Denan District Health Bureau report). Awareness-creation and sensitization activities performed in relation to malaria control, the availability of anti-mosquito repellents in most drug shops, and the culture of the community to use plant parts for different smoking activities may have contributed to practices towards the control and prevention of VL in our study area.

Along with the sero-prevalence and KAP studies, an entomological investigation was carried out to determine the sand fly vectors of VL. Among the 65 sand fly species known in Ethiopia, 12 (18.5%) species of sand flies were identified in this study. The sand fly species composition recorded in this study is consistent with previous reports in other parts of Ethiopia [11, 19, 40, 41]. Five species of *Phlebotomus* were identified during this study, with *P. orientalis* being the dominant species, constituting 11.42% of total sand fly captures. *P. orientalis* is the vector of VL caused by *L. donovani* in Sudan, South Sudan [42, 43], southwestern Ethiopia [16], and northern Ethiopia [18]. Apparently, this sand fly species could be involved in the transmission of VL in this VL focus. Therefore, further studies to determine the infection rates and host preference patterns of this vector species are required.

Another epidemiologically important species of the genus *Phlebotomus* found during our surveys included *P. alexandri, P. papatasi, P. rodhaini, and P. saevus*. The vectorial status of *P. alexandri* is not clearly established in different parts of Ethiopia, despite the fact that it is a proven vector of *L. infantum*, causative of zoonotic VL in China [44]. *P. rodhaini* reported in this study is also suspected as a VL vector in eastern Sudan and is implicated in maintaining the zoonotic cycle between reservoir animals [45]. *L. tropica* was also isolated from two specimens of *P. saevus* in the Awash Valley, northeastern Ethiopia [46]. So far no clinically confirmed cases of CL were reported in health centers in the district (Head of Health Bureau, personal communication). Similarly, *P. papatasi* is the principal vector of *L. major* in most parts of the Old World [47], although its vectorial role in CL transmission in Ethiopia is yet unclear.

Regarding microhabitat preference, most of the *P. orientalis* were captured outdoors. Such a higher abundance of this species in outdoor habitats could be related to the greater availability of suitable resting and breeding habitats. In addition, putative habitats of sand fly resting and breeding areas, including termite mounds, cracked walls, and animal enclosures in peri-domestic ecotopes were seen in all of the study kebeles. Studies also confirm that these habitats are known to contribute to an increased density of blood-questing sand flies [48]. Importantly, residents in the study area practice outdoor sleeping close to animal enclosures, suggesting that these habitats could be probable areas where people get VL infections.

Limitations of the study design and the methods of data collection might create some potential for bias in this study. The cross-sectional design of the study, with a focus on recruiting participants who sought healthcare, might have influenced their knowledge and practices in VL control. In addition, there are some gaps, such as infection rates in sand flies and blood meal sources, which deserve to be addressed in another entomological study in this particular VL focus by targeting those issues.

## Conclusions

Our results suggest the occurrence of asymptomatic VL infections in Denan district, which is a new VL focus. Adults in the age group 19–36 showed higher VL sero-reactivity compared to other age groups in the study. Factors associated with an increased risk of VL infection were living in houses with damp floors, outdoor sleeping behaviors, and sleeping near an animal shelter. Poor knowledge coupled with unfavorable attitudes and practices towards the disease are also alarming for the control of VL, and this further entails the need to increase awareness of VL transmission and prevention in pastoral communities. *P. orientalis*, a proven vector of VL, appears to play a role in the transmission of VL in this new VL focus. Further in-depth epidemiological and molecular studies and investigations of other contributing risk factors in the area are warranted. Further research is recommended on host preferences and *Leishmania* infection rate in *P. orientalis* in this VL focus.

## Data Availability

The datasets generated during and/or analyzed during the current study are available from the corresponding author on reasonable request.

## Abbreviations

CDC-LTs: CDC light traps
CI: Confidence interval
DAT: Direct Agglutination Test
ELISAs: Enzyme linked immunosorbent assays
KAP: Knowledge, attitudes and practices
ORs: Odds ratios
ST: Sticky traps
VL: Visceral leishmaniasis
